# Decolonising global health: why the new Pandemic Agreement should have included the principle of subsidiarity

**DOI:** 10.1016/S2214-109X(24)00186-4

**Published:** 2024-05-09

**Authors:** Thana C de Campos-Rudinsky, Sarah L Bosha, Daniel Wainstock, Sharifah Sekalala, Sridhar Venkatapuram, Caesar Alimsinya Atuire

**Affiliations:** Escuela de Gobierno, Pontifical Catholic University of Chile, Santiago, Chile; O’Neill Institute for National and Global Health Law, Georgetown University Law Center, Washington, DC, USA; Pontifical Catholic University of Rio de Janeiro, Rio de Janeiro, Brazil; School of Law, University of Warwick, Coventry, Warwickshire, UK; Global Health Institute, Population Health Sciences, King’s College London, London, UK; Department of Philosophy, University of Johannesburg, Johannesburg, South Africa; Centre for Tropical Medicine and Global Health, Nuffield Department of Medicine, Old Road Campus, Oxford, UK; University of Ghana, Accra, Ghana

## Abstract

The negotiations for the WHO Pandemic Agreement have brought attention to issues of racism and colonialism in global health. Although the agreement aims to promote global solidarity, it fails to address these deeply embedded problems. This Viewpoint argues that not including the principle of subsidiarity into Article 4 of the agreement as a pragmatic strategy was a missed opportunity to decolonise global health governance and promote global solidarity. Subsidiarity, as a structural principle, empowers local units to make decisions and address issues at their level, fostering collaboration, coordination, and cooperation. By integrating subsidiarity, the agreement could have ensured contextually appropriate responses, empowered local communities, and achieved justice in global health. This paper discusses the elements of subsidiarity—namely, agency and non-abandonment—and highlights the need to strike a balance between them. It also maps the principle of subsidiarity within the Pandemic Agreement, emphasising the importance of creating a practical framework for its implementation. By integrating subsidiarity into the agreement, a just and decolonialised approach to pandemic prevention and response could have been closer to being realised, promoting global solidarity and addressing health inequities.

## Introduction

The WHO Pandemic Agreement (previously known as the WHO Pandemic Treaty) is being negotiated in a world where racism and the lingering colonial impacts on global health remain prominent.^1^ As COVID-19 highlighted, many health inequities, such as those associated with race and geographical regions, have not been addressed and global solidarity, particularly in the form of equitable vaccine distribution, remains unrealised.^2–4^ The Pandemic Agreement aims to promote global solidarity through the collaboration and cooperation of governments on a local and international level to ensure fair, equitable, and prepared responses to pandemics.^5^ However, the agreement does not address the issues of racism and colonialism in global health. Indeed, health actors have argued that equity cannot be achieved in global health governance without an intentional focus on decolonising actions that recognises the deeply embedded racist practices and colonial structures that still exist.^6^

We argue that the failure to include the principle of subsidiarity into Article 4 of the Pandemic Agreement as a pragmatic strategy was a missed opportunity to decolonise global health governance and promote global solidarity. Although the zero draft of the document released on Feb 1, 2023,^5^ encompassed various principles, some of which might touch on aspects of subsidiarity, we contend that explicitly incorporating subsidiarity is crucial to ensure a comprehensive and coherent approach to pandemic prevention, preparedness, and response within the framework of global solidarity.

Solidarity, a fundamental human rights principle,^7^ is legally defined as promoting unity among individuals, communities, states, and international organisations to achieve common goals. Subsidiarity, a structural principle of justice, suggests that decision-making authority primarily lies with the level of governance closest to the issue or the community needs.^8,9^ Local solutions are preferred whenever possible before involving high levels of authority, thus favouring a bottom-up approach.^10–14^ Subsidiarity, therefore, ensures collaboration, coordination, and cooperation among stakeholders, holding everyone accountable for the wellbeing of the entire community, without leaving anyone behind.^14,15^

By empowering small units to actively participate in decision making, subsidiarity enables informed and contextually appropriate responses to a pandemic. It recognises that local communities have a deeper understanding of their own circumstances than high levels of authority and they can contribute valuable knowledge and expertise.^16^ This approach also fosters a sense of ownership and responsibility among community members as they are actively involved providing solutions that affect their lives.^9,17,18^ By restoring agency within the context of tailored assistance driven by community needs, subsidiarity provides practical means to decolonise global health governance.

### Defining the elements of subsidiarity

Integrating the principle of subsidiarity into the new Pandemic Agreement would foster contextually adequate solutions to the challenges posed by pandemics. This approach seeks to avoid the criticised practice of imposing one-size-fits-all prescriptions by centralised global organisations.^15^ A decentralised approach reduces the reliance on a small number of powerful, private, and public actors who have substantial control over national governments, civil society organisations, and non-governmental organisations.^15,19^

The principle of subsidiarity is comprised of two essential elements—non-abandonment and agency—which are both indispensable for achieving justice.^15^ Finding the right balance between these elements is crucial to prevent negative consequences. Over-emphasising non-abandonment while disregarding agency might lead to patronising forms of assistance. Conversely, prioritising agency alone, as seen in instances of vaccine nationalism and hoarding, encourages an individualistic approach that hampers collaboration, coordination, and cooperation among communities. This strategy ultimately undermines efforts to foster global solidarity.

The element of agency empowers local units with the freedom and responsibility to use their own knowledge and expertise as a primary resource.^9,20^ Communities are empowered to identify when and what type of assistance they require from other units in a collaborative effort, which enables a decolonial approach. The specific moral wrong of colonialism is defined as the subtraction of agency of communities and people, when their agency is removed in the service of the interests of colonisers.^9^ Unlike mainstream approaches that might superficially engage with community participation, subsidiarity fosters genuine empowerment by recognising the agency and expertise of local actors in identifying and addressing health needs. By devolving authority at the local level, subsidiarity facilitates meaningful partnerships between governments, communities, and civil society organisations, amplifying their voices in shaping health policies and interventions.

The HIV pandemic serves as a prominent example of when agency has been harnessed to reform global health policy, particularly in disease prevention and treatment. Community activists, including people living with HIV, became partners and collaborators rather than constituents or study participants, fundamentally altering the course of research and treatment approaches.^21^

The element of non-abandonment works alongside agency by ensuring that local authorities can exercise their autonomy while receiving support when needed from bigger-level units. Local units are, therefore, provided with assistance on explicit request, which is tailored to their specific needs and preferences, rather than being dictated by the agendas of other countries, market forces, or global health donors.^22,23^ Additionally, corrupt local units are not left free to use the aid for fraudulent, self-serving purposes. Although the agency of local governments should be respected, the voice of civil society organisations and non-governmental organisations is key in the fight against a corrupt government. By incorporating the principle of subsidiarity into the Pandemic Agreement, the importance of focusing on the needs of local communities and respecting their autonomy in decision making are reinforced, emphasising their duty to assist while empowering them to define the type of assistance they require.

### Recommendations for global health governance

To effectively address the gaps in the Pandemic Agreement and uphold the principle of subsidiarity within global health governance, it is essential to adopt a language that empowers local communities, enhances agency, and promotes inclusive decision making at all levels. These recommendations serve as crucial steps towards fostering a more equitable and participatory global health system. Therefore, we believe that the Pandemic Agreement should include the following mechanisms.

#### Equal voting powers and decision-making authority

To uphold the principle of subsidiarity, it is important to grant equal voting powers and decision-making authority to all units of the governing body, including community-based civil society organisations. Currently, the Pandemic Agreement primarily grants decision-making power to national governments, whereas other local community units, such as civil society organisations, have advisory roles without voting powers. This disparity hampers inclusivity and effective compliance monitoring. Following the example of the International Labor Organization, which grants voting rights to its tripartite members (eg, national governments, workers unions, and employers), the governing body should adopt an inclusive decision-making structure that ensures meaningful participation of all relevant stakeholders.^24^

#### Strengthening community engagement

The Pandemic Agreement proposes that small-level units should engage in agreement monitoring through the consultative body and provide advice on community responses to pandemics. However, this recommendation could be further strengthened by explicitly recognising and promoting community engagement at all stages of decision making. It is essential to involve local communities in the design, implementation, and evaluation of policies and interventions as they possess invaluable knowledge and insights about their situational context.^25^ If diverse communities, such as non-governmental organisations, women’s groups, faith groups, and other groups representing marginalised populations, actively participate in agreement monitoring, their collective wisdom and expertise can be accessed to enhance the effectiveness of global health governance.^19^

#### Enhancing mechanisms for community redress and accountability

It remains unclear what enforcement mechanisms the Pandemic Agreement will take, which raises concerns about the risk of both subtraction of local agency by top-down impositions and abandonment of communities affected by non-compliance and the capacity to lodge complaints. Any complaint mechanisms should go beyond state parties and ensure that affected communities can seek redress and accountability. For instance, the First Optional Protocol allows individuals to submit a complaint about state parties to the UN Human Rights Committee; the African Commission on Human and Peoples Rights, which receives communications on agreement violations of the African Charter on Human and Peoples Rights, also permit individual complaints. Such a system can be achieved by partnering with existing regional bodies or creating in-country mechanisms that allow affected communities to seek redress and provide feedback on the effect of the agreement on their local operations. By facilitating community participation in decision-making processes and providing avenues for grievance redressal, the agreement can ensure that the element of non-abandonment is upheld and the voices of affected communities are heard.

#### Subsidiarity-sensitive impact assessments

The Pandemic Agreement should include provisions for subsidiarity-sensitive impact assessments to evaluate the potential effects of global health policies and interventions at different levels, particularly at the local and community levels. These assessments should consider the specific contexts, capacities, and needs of communities, ensuring that interventions are tailored and responsive to local realities. By integrating subsidiarity-sensitive impact assessments into the decision-making process, the agreement can promote targeted and effective global health governance while respecting the diversity and autonomy of local communities.

Additionally, efforts should be made to strengthen decentralised governance structures, promote local ownership, and build capacities at the basic level. These targets can be achieved through fostering partnerships between global, regional, and local actors, investing in community-led initiatives, and promoting knowledge exchange and learning across different levels of governance.^18^ By complementing agreement-based approaches with these endeavours, global health governance can embrace the principles of subsidiarity and ensure that decisions are made at the most appropriate and effective levels, leading to equitable and sustainable health outcomes for all.

#### Strengthening health workforce management

Subsidiarity can revolutionise the approach to managing the health workforce. Traditional top-down approaches often do not consider the diverse needs and contexts of local communities, resulting in mismatches between available health-care resources and community needs.^15^ By embracing subsidiarity, decision-making authority can be decentralised to local health establishments, empowering them to tailor workforce development strategies according to the specific health challenges and cultural nuances of their communities. For instance, in regions with severe shortages of health-care professionals, local authorities could implement training programmes to upskill community members as health-care workers. By decentralising decision making on workforce management and training initiatives, the Agreement can ensure that health-care services are accessible and responsive to local needs, thus ultimately improving health outcomes in underserved areas.

#### Reconsidering surveillance systems

Subsidiarity can play a pivotal role in redesigning surveillance systems by leveraging local knowledge and resources to improve disease detection and response. Centralised surveillance systems might overlook local outbreaks or fail to capture real-time data due to bureaucratic delays. For example, community health workers can be trained to recognise the early signs of outbreaks and report them to local health authorities. By decentralising surveillance efforts and involving local health authorities, communities can effectively monitor disease trends, respond rapidly to emerging threats, and implement targeted interventions. Surveillance should be implemented, whenever possible, by the local community (not by WHO) as a bottom-up approach, allowing for a more participatory, efficient, and responsive strategy to the specific needs of each community. This approach not only enhances the timeliness and accuracy of surveillance data but also builds trust between communities and health authorities, facilitating information sharing and collaboration in disease control efforts.

#### Advancing One Health approaches

The principle of subsidiarity aligns with One Health approaches, which recognise the interconnectedness of the health of humans, animals, and the environment. Local communities are often at the forefront of interactions between humans, animals, and the environment, making them key stakeholders in One Health initiatives. For example, in regions where zoonotic diseases are prevalent, community-based surveillance programmes can monitor animal health and detect potential spillover events. By involving local communities in One Health initiatives, the agreement can promote holistic approaches to disease prevention and control, ultimately improving health outcomes for humans and animals.

## Conclusion

It is imperative to clarify the scope of our discourse and address potential implications associated with the principle of subsidiarity within the context of the Pandemic Agreement. Although our objective is to advocate for the integration of subsidiarity into the Agreement framework to enhance global health governance, it is essential to delineate the boundaries of our argument. Our advocacy for subsidiarity does not automatically imply an absolute removal of specific mandates outlined within the Agreement, such as those pertaining to core commitments to pandemic preparedness. Instead, our emphasis lies in highlighting subsidiarity as a structural principle of justice aimed at empowering local communities while ensuring accountability and equity within the broader structures of global health governance. The Agreement should be built on the premise that only through perceiving the world as one community, standing in solidarity, can the threat that pandemics pose to lives and livelihoods be successfully targeted. We invite further deliberation and exploration into effectively managing tensions between rights, equity, and subsidiarity within the context of global health governance.
